# Hospital exemption use of virus-specific T-lymphocytes in children: early experience within the Allovista clinical trial framework

**DOI:** 10.3389/fimmu.2026.1805803

**Published:** 2026-04-10

**Authors:** Marek Ussowicz, Aleksandra Ślęzak, Marta Myszka-Kozłowska, Kornelia Gajek, Karol Jopek, Marta Bukowska, Alicja Bukowska, Justyna Jureczek, Wioletta Jarmużek, Mieczysław Litwin, Maksymilian Deręgowski, Katarzyna Derwich, Anna Czyż, Anna Bogacz

**Affiliations:** 1 Department of Paediatric Bone Marrow Transplantation, Oncology and Hematology, Wroclaw Medical University, Wrocław, Poland; 2 Department of Histology and Embryology, Poznan University of Medical Sciences, Poznan, Poland; 3 Department of Personalized Medicine and Cell Therapy, Regional Blood Center, Poznan, Poland; 4 Division of Histology and Embryology, Department of Human Morphology and Embryology, Wroclaw Medical University, Wrocław, Poland; 5 Department of Nephrology, Kidney Transplantation and Hypertension, Children’s Memorial Health Institute, Warsaw, Poland; 6 Department of Pediatric Oncology, Hematology and Transplantology, Poznan University of Medical Sciences, Poznań, Poland; 7 Department and Clinic of Hematology, Cellular Therapies and Internal Medicine, Wroclaw Medical University, Wrocław, Poland; 8 Department of Cancer Immunology, Poznan University of Medical Sciences, Poznan, Poland

**Keywords:** adenovirus, adoptive T-cell therapy, allogeneic hematopoietic stem cell transplantation, Epstein–Barr virus, pediatric, post-transplant lymphoproliferative disorder, refractory viral infection, virus-specific T cells

## Abstract

**Background:**

Adoptive transfer of virus-specific T lymphocytes (VST) is an emerging strategy for refractory viral infections in immunocompromised individuals and is being prospectively evaluated in the ALLOVISTA clinical trial. Here we describe our experience with hospital-exemption advanced therapy medicinal product (ATMP-HE) VST in six pediatric transplant recipients.

**Methods:**

Six children and adolescents (median age 4.5 years, range 2.5–18.5) treated in two Polish centers received VST for refractory viremia after allogeneic hematopoietic stem cell transplantation: adenovirus (ADV) in three cases, EBV-driven disease (EBV/PTLD or HL relapse with EBV reactivation) in three, including one patient with concomitant CMV/EBV/ADV infection. All patients had failed conventional antiviral and/or immunochemotherapy (cidofovir, rituximab, chemotherapy). VST were generated from allo-HSCT or related donors using automated CliniMACS Prodigy Cytokine Capture System (IFN-γ CCS, Miltenyi Biotec) following antigenic stimulation with viral peptide pools and released as ATMP-HE according to the ALLOVISTA protocol. A single intravenous infusion of 1.01×10^5^–1.85×10^6^ VST (median 2.5×10^5^) was administered. Virological response was assessed by quantitative PCR up to 4 weeks post-infusion, acute GVHD (aGVHD) and survival were recorded.

**Results:**

Virological outcomes were evaluable in five patients. Four of five (80%) evaluated patients achieved at least a 1-log reduction in viral load, including two (40%) with complete clearance by week 4 (one EBV, one ADV). Two additional patients had marked but incomplete or transient viral load reductions, one patient showed minimal virological change. Despite biological activity, four children died: from progressive EBV disease (n=1), ADV infection (n=1), relapse of HL (n=1) and multi-organ failure with invasive fungal disease in a child with multi-viral infection (n=1). Two patients with complete virological responses are alive with sustained remission of viremia at the last follow-up. No new-onset or worsening aGVHD was documented after VST infusion.

**Conclusions:**

VST administered as an ATMP-HE are feasible in routine clinical practice and demonstrate rapid antiviral activity in heavily pretreated pediatric patients. However, advanced viral disease and competing complications limit overall survival, underscoring the need for earlier intervention and advocating for treatment within the ongoing ALLOVISTA trial.

## Introduction

Refractory viral infections remain a major cause of morbidity and mortality after allogeneic hematopoietic stem cell transplantation (allo-HSCT), particularly when pharmacologic antivirals are ineffective, poorly tolerated or contraindicated. Adoptive transfer of virus specific T-lymphocytes (VST) has emerged as a salvage strategy because it directly reconstitutes antiviral cellular immunity and can be used against cytomegalovirus (CMV), Epstein-Barr virus (EBV), adenovirus (AdV), and BK polyomavirus among other pathogens (1, 2). In principle, infused antigen-experienced VST can traffic to sites of infection, undergo antigen-driven activation and expansion *in vivo*, and contribute to viral clearance while supporting the longer-term reconstitution of virus-specific immune memory. Deep sequencing-based tracking studies have demonstrated that adoptively transferred virus-specific T-cell clonotypes can expand after antigen encounter and persist for months, supporting a mechanistic link between cellular engraftment and clinical effect (3, 4).

We present early real-world data within the ALLOVISTA clinical trial framework regarding donor-derived, IFN-γ-captured VST administered as a hospital-exemption Advanced Therapy Medicinal Products (ATMP-HE) to six pediatric transplant recipients suffering from refractory ADV viremia and EBV-related diseases, including posttransplant lymphoproliferative disease (EBV-PTLD) or EBV-associated recurrence. The six pediatric patients described in this report were treated exclusively under this ATMP-HE pathway and did not participate in the formal ALLOVISTA trial.

### Allovista clinical trial overview

ALLOVISTA (ALLOgeneic Virus-Specific T-cell Adoptive therapy) is a multicenter, open-label, single-arm Phase I dose-escalation clinical trial (funded by Polish Medical Research Agency, 2020/ABM/01/00125) sponsored by Wroclaw Medical University (Uniwersytet Medyczny im. Piastów Śląskich we Wrocławiu). The study is registered in CTIS under EU CT 2024-517696-21-01 (5). The primary objective is to evaluate safety and determine the maximum tolerated dose (MTD) of adoptive cellular therapy with CMV specific T lymphocytes (VST_RCKiK) administered to adult recipients after allo-HSCT with refractory CMV infection. VST_RCKiK is produced from non-mobilized donor leukapheresis, processed in a GMP (Good Manufacturing Practice)/ATMP (Advanced Therapy Medicinal Product) facility (RCKiK: ‘Regionalne Centrum Krwiodawstwa i Krwiolecznictwa’, i.e., Regional Blood Donation and Blood Treatment Center in Poznań, Poznań, Poland) using the CliniMACS Prodigy Cytokine Capture System. Briefly, the system enriches virus antigen-reactive T cells by stimulating donor leukocytes with virus-specific peptide pools, then immunomagnetically capturing cells that secrete interferon gamma (IFN-γ) in response — yielding a polyclonal, antigen-experienced T-cell product enriched for both CD4^+^ and CD8^+^ VST cells. The study uses a standard 3 + 3 design with three escalating dose levels (1×10^4^–5×10^4^/kg; 5×10^4^–1×10^5^/kg; 1×10^5^–5×10^5^/kg IFN-γ–secreting CMV-specific T cells.

VST products are preferentially manufactured from the original allo-HSCT donor; a third-party donor registry comprising approximately 500 healthy blood donors (recruited with the help of the Regional Blood Donation and Blood Treatment Center in Wrocław, Poland), with documented HLA typing and viral reactivity profiles, serves as a resource for cases where the original donor is unavailable or non-reactive. Pending final regulatory decisions, the ALLOVISTA clinical trial framework provides VSTs targeting CMV, EBV, adenovirus, and polyomaviruses under a hospital exemption pathway.

## Methods

### Manufacture of donor-derived virus-specific T cells

Before manufacturing, donor reactivity was confirmed using an IFN-γ release assay (Miltenyi Biotec) with the same MACS GMP PepTivator peptide pools (AdV Select or EBV Select, as appropriate) that would be used in production, ensuring only seropositive, reactive donors proceeded to cell manufacture. Donor-derived VSTs directed against ADV or EBV were generated from leukocytes using a rapid cytokine-capture approach on the CliniMACS Prodigy platform (CCS, IFN-γ; Miltenyi Biotec) in a closed, automated workflow (6). The CCS IFN-γ enrichment process accepts starting material in the range of 50–150 mL with a maximum capacity of 1×10^9^ white blood cells. The employed MACS GMP PepTivator peptide pools were MACS GMP PepTivator AdV Select (ref. 170-076-169, Miltenyi Biotec) for adenovirus-targeted VST and MACS GMP PepTivator EBV Select (ref. 170-076-143, Miltenyi Biotec) for EBV-targeted VST. PepTivator pools consist predominantly of overlapping 15-mer peptides (typically with 11-amino-acid overlap), supporting activation of both CD4+ and CD8+ antigen-specific T cells (7). PepTivator products were reconstituted in 8 mL sterile water for injection and transferred via a sterile filter to the Prodigy antigen bag when prompted by the software.

### Automated IFN-γ capture and immunomagnetic enrichment

Following antigen stimulation, the CliniMACS Prodigy CCS IFN-γ workflow proceeds through sequential phases: sample loading, antigen stimulation, catchmatrix labeling, secretion phase, enrichment reagent labeling, washing, magnetic separation, and elution. The typical runtime was approximately 12 hours (without delay), and the full process was always completed within 24/36 hours since apheresis start. The process buffer was prepared by supplementing CliniMACS PBS/EDTA buffer with human serum albumin (HSA) to a final concentration of 0.5% (w/v), as specified in the CCS IFN-γ user manual. Target cells (IFN-γ–positive fraction) were eluted at the end of the run in approximately 7–8 mL of elution buffer (0.9% sodium chloride infusion solution), with HSA supplementation per protocol. In-process QC sampling was performed according to the automated workflow. Product release and administration proceeded per institutional GMP release specifications. Product release criteria included: viability ≥70% (automated cell counting), IFN-γ^+^ T cell fraction by flow cytometry (efficacy surrogate), sterility (Gram stain, endotoxin assay), T cell identity (CD3^+^ fraction), and mycoplasma exclusion by PCR. The automated workflow runtime was approximately 12 hours, and products were released and administered within 36 hours from the start of apheresis in all cases. Each patient received a single intravenous infusion. The administered dose ranged from 1.01×10^5^ to 1.85×10^6^ VST (median 2.5×10^5^).

### Patient characteristics

Six pediatric allo-HSCT recipients were treated in two Polish centers. Patients’ clinical details are presented in **Table 1**. Median age at VST infusion was 4.5 years (range 2.5–18.5). The underlying conditions were hemophagocytic lymphohistiocytosis (HLH, n=2), severe aplastic anemia (SAA, n=1), chronic granulomatous disease (CGD, n=1), Hodgkin lymphoma (HL, n=1) and acute myeloid leukemia (AML, n=1). Indications for VST were refractory adenovirus (ADV) viremia (n=3) and EBV-driven disease (n=3, EBV/PTLD or EBV-associated HL relapse), including one patient (P4) with concomitant CMV/EBV/ADV infection. Despite concomitant CMV reactivation, CMV-specific VST were not manufactured for this patient for two reasons. First, the CliniMACS Prodigy CCS IFN-γ platform permits pooling of a maximum of two PepTivator peptide pools per manufacturing run. Second, CMV viremia was under pharmacological control with ganciclovir at the time of the VST decision; the two uncontrolled and clinically dominant threats — high-level ADV replication and EBV-driven lymphoproliferation — were therefore selected as the stimulation targets. For P1 (post-kidney transplant) PTLD was diagnosed as diffuse large B-cell lymphoma (DLBCL), WHO grade III, with multifocal involvement (cervical lymph nodes, tonsils, and mesenteric nodes) confirmed by PET-CT and cervical node biopsy. For P6 (post-allo-HSCT for AML) PTLD was polymorphic PTLD with EBV-positive DLBCL features, localized to mediastinal nodes, confirmed by CT and biopsy. All patients had failed conventional antiviral therapy before VST infusion (including cidofovir, rituximab and/or chemotherapy, as applicable). In all six patients, immunosuppressive therapy was discontinued prior to VST infusion to facilitate T-lymphocyte expansion and engraftment.

**Table 1:** Patient characteristics, VST treatment, and outcomes.

Patient	Diagnosis	Transplantation history	Indication	Infection onset (post HSCT/SOT days)	Days from infection to VST	Lymphocyte counts/µL prior to VST infusion	Viral load pre-VST IU/mL	Prior therapy	VST dose (×10^5)/kg BW	Viral load wk1 IU/mL	Viral load wk4 IU/mL	aGVHD	Outcome
P1	HLH	Post kidney transplantation	EBV-associated PTLD	1954	124	0	60000000	Rituximab, chemotherapy CHOP.	1.01	7278	520563	No	Death: Progressive EBV disease
P2	SAA	Matched sibling donor allo-HSCT	Refractory ADV viremia	40	32	0	18881	Cidofovir ×3	1.50	4446	NA	No	Death: Progressive ADV disease
P3	GCD	Haploidentical donor allo-HSCT	Refractory ADV viremia	4	44	CD3: 377; CD4: 30; CD8: 230; NK: 274	120000	Cidofovir ×7	2.50	11500	0	No	Alive
P4	HLH	Matched unrelated donor allo-HSCT	Refractory ADV viremia	69	72	CD3: 89; CD4: 18; CD8: 60; NK: 7	194000	NA	4.00	3800	NA	No	Death: Multi-organ failure; invasive fungal disease, multi-viral infection (CMV, EBV, ADV)
			EBV-viremia	57	90		40100	Rituximab ×6		13100	NA		
P5	HL	Haploidentical donor allo-HSCT	EBV-associated HL relapse	515	118	CD3: 19; CD4: 6; CD8: 14; NK: 21	359,000	Chemotherapy, immunotherapy	18.50	NA	NA	No	Death: Progressive Hodgkin lymphoma
P6	AML	Matched unrelated donor allo-HSCT	EBV-associated PTLD	33	37	n/a	12,000	Rituximab ×4	2.50	0	0	Pre-existing (grade 2)	Alive

ADV, adenovirus; aGVHD, acute graft-versus-host disease; AML, acute myeloid leukemia; CMV, cytomegalovirus; EBV, Epstein–Barr virus; GCD, chronic granulomatous disease; HL, Hodgkin lymphoma; HLH, hemophagocytic lymphohistiocytosis; HSCT, hematopoietic stem cell transplantation; n/a, not available; NK, natural killer cells; PTLD, post-transplant lymphoproliferative disorder; SAA, severe aplastic anemia; SOT, solid organ transplantation; VST, virus-specific T cells.

VST products were manufactured from haploidentical donors in all cases; original haploidentical allo-HSCT donors (n=2, patients 3 and 5) or biological parents (n=4, remaining patients).

## Results

**Figure 1:**
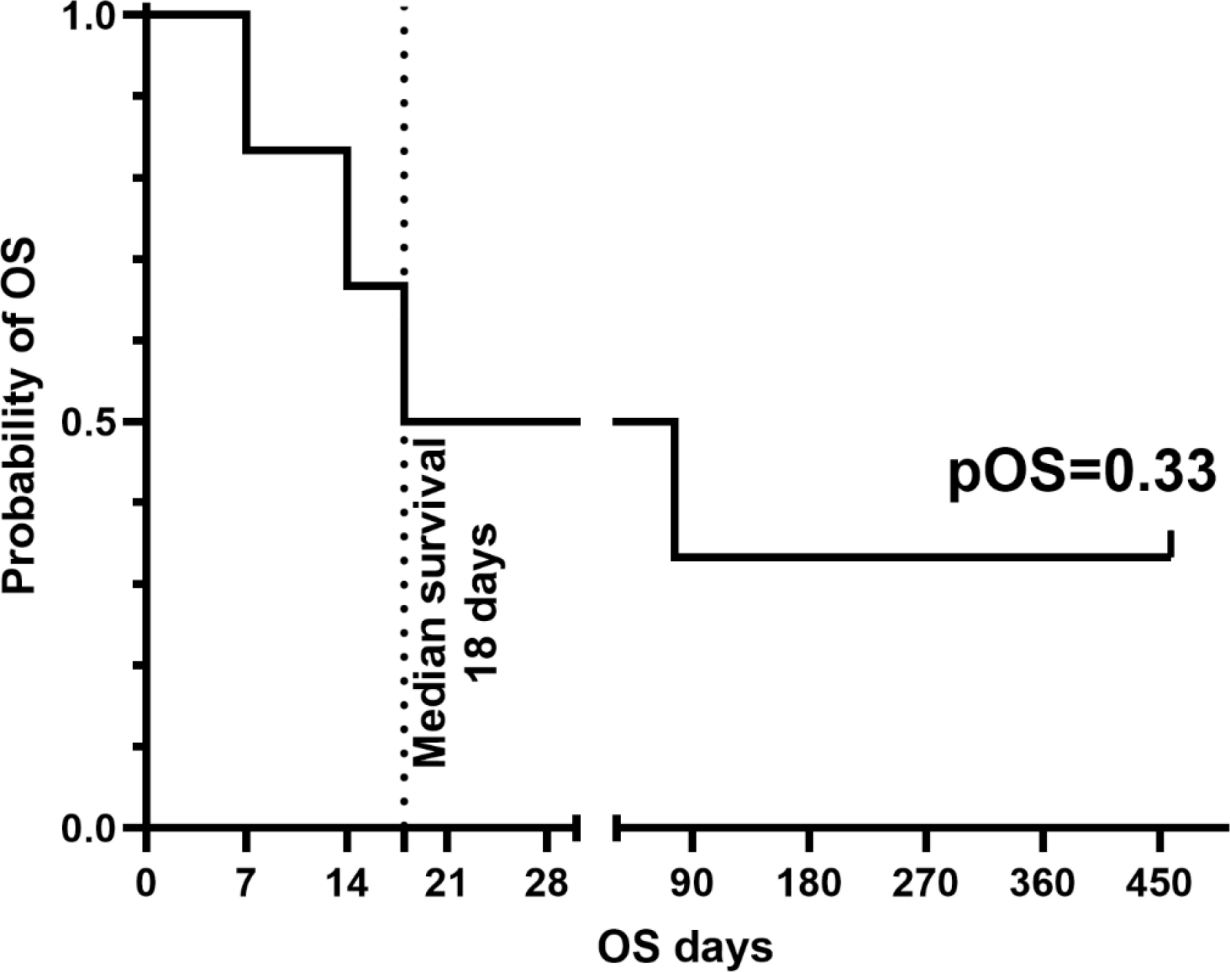
Overall survival (OS) following VST infusion.

Virological response by quantitative PCR was evaluable in five of six patients. Four of five (80%) achieved at least a 1-log reduction in viral load within 4 weeks. Complete viral clearance by week 4 was documented in two patients (40% of evaluable patients): one with EBV-driven disease and one with ADV infection. Two additional patients demonstrated marked but incomplete or transient viral load reductions. One patient showed minimal virological change during the 4-week post-infusion observation window. Systematic immunological monitoring was not available for the two surviving patients. Despite evidence of biological antiviral activity, four patients died from progressive EBV disease (n=1), persistent/progressive ADV infection (n=1), relapse of the underlying malignancy (HL, n=1), or multi-organ failure with invasive fungal disease in a patient with multi-viral infection (n=1). The two patients with complete virological responses (P3 and P6) remain alive and PCR-negative 16 months post VST infusion, with no PTLD recurrence or ADV reactivation detected (**Figure 1**). No new-onset or worsening acute graft-versus-host disease (aGVHD) was observed following immunosuppression withdrawal or VST administration in any patient, which is noteworthy given that all donors were haploidentical. No infusion-related reactions, including fever, chills, hypotension, or bronchospasm, were observed during or within 24 hours of any infusion.

## Discussion

A rapid biological response was seen in this extensively pretreated population. Of the evaluable patients, 80% achieved a minimum 1-log reduction in viral load within 4 weeks, with full eradication observed in two cases by week 4. The response kinetics align with the predicted mechanism of antigen-mediated activation and *in vivo* proliferation of infused VSTs.

In our cohort, virological response was associated with survival, whereas patients with late-stage disease and progressive end-organ dysfunction frequently did not respond despite VST administration. This observation parallels the findings of Neller et al., where patients with late-stage progressive disease and persistently high viral loads at the time of VST infusion showed no evidence of response, reinforcing argument that earlier referral, before irreversible end-organ dysfunction accumulates, is critical for meaningful clinical benefit (8). Similar observations have been reported in early clinical experience, where delayed infusion in critically ill patients was linked to poor response rates, while earlier intervention was associated with durable clinical benefit (9, 10). Nonetheless, antiviral efficacy did not result in improved overall survival for the majority of patients. It must be pointed out, that VSTs were administered as a therapeutic experiment reserved for individuals with no remaining standard treatment options- typically profoundly ill, heavily pretreated, and with advanced viral disease at the time of infusion.

VST infusions were well tolerated in our cohort with no acute GVHD and no treatment-related mortality. Although third-party products are often partially HLA-mismatched, clinical experience indicates that appropriately selected virus-specific T cells are generally safe and do not trigger clinically significant GVHD, even when some *in vitro* alloreactivity can be demonstrated (11–14). While the safety profile of partially HLA-matched third-party VST is well-documented, questions regarding efficacy and *in vivo* persistence remain. Our experience aligns with the findings of Bonifacius et al., who demonstrated that EBV-specific CTL products manufactured from stem cell donors, related third-party donors, and unrelated registry donors are all clinically effective and well-tolerated, including in patients with compromised organ function (15). Importantly, in the largest head-to-head comparison to date specifically in children and young adults, Galletta et al. found no statistically significant difference in virological response rate between donor-derived and third-party VST products, supporting the feasibility of both approaches in the pediatric setting (16). Third-party products, by nature of HLA restriction, may undergo faster immune-mediated elimination by the host, limiting antigen-driven expansion and durable control of viral replication. The recent discontinuation of posoleucel’s clinical development program by AlloVir — despite earlier promising phase 2 results — highlights the challenges of demonstrating efficacy for partially matched off-the-shelf VST in randomized controlled trials, and emphasizes the potential advantage of related and better matched donor-derived VST as used in our cohort (17).

Nonetheless, infusion-related reactions and complications, including GVHD, cytokine release syndrome, pulmonary problems such as transfusion-related acute lung injury (TRALI), thrombotic microangiopathy, and graft rejection, are recognized as dangers of cellular treatment and require careful monitoring in larger prospective cohorts. The lack of GVHD signs in our limited cohort is encouraging but does not rule out the possibility of uncommon, delayed, or clinically complicated immune-mediated adverse effects (18, 19). Previous clinical experience demonstrates that gastrointestinal symptoms occurring concurrently with VST expansion may signify virus-specific tissue infiltration instead of traditional GVHD, emphasizing the necessity for careful evaluation in prospective protocols (2).

It must be pointed out, that VSTs are designed to offer a temporary restoration of antiviral immunity, with donor-derived VST often persisting relatively briefly (typically for up to 30 and generally not longer than approximately 90 days), however instances of prolonged persistence have also been documented (3, 4, 10, 20, 21). Our findings emphasize the significance of timing and patient selection. When VSTs are administered late, following prolonged high-level viremia, established organ disease, or in the patients with severe immune dysregulation, even swift reductions in viral load might be insufficient to prevent severe organ damage or fatal secondary outcomes. Early administration, prior to the progression to multi-organ involvement, may be essential for turning antiviral efficacy into a therapeutic advantage.

Scalability, rapid manufacturing and access to ATMPs remain key factors for real-world efficacy. Recent experience supports automated and semi-automated, closed-system VST manufacturing with shortened vein-to-vein times (22, 23). Moreover third-party bank models have demonstrated the feasibility of providing partially HLA-matched products on demand (24). From a manufacturing perspective, rapid selection strategies based on short antigen stimulation followed by INF-γ cytokine capture enrich antigen-reactive T cells while limiting the proportion of alloreactive cells, enabling timely access to therapy in urgent settings (9, 22, 25, 26). It exemplifies how closed, fully automated benchtop systems can enable ATMP production with reduced facility requirements compared to traditional open manufacturing, potentially allowing centers with standard GMP cleanroom infrastructure, rather than large-scale manufacturing suites, to produce VST products (21, 22). Such approaches may succeed as hospital exemption manufacturing by improving coverage, shortening lead times, and facilitating broader access where individualized donor-derived products are not feasible (13, 17, 23, 26, 27). Our hospital-exemption experience illustrates the organizational capability of the entire ALLOVISTA clinical trial framework, to treat patients under a hospital exemption pathway and within clinical trial.

Several limitations must be acknowledged. The cohort is limited in size and diverse regarding underlying disorders, post-transplant time, type and severity of viral infection, as well as concomitant immunosuppression and supportive therapy. Virological outcomes were not measurable in all patients, response evaluation was confined to early post-infusion time intervals, and immunological monitoring was not standardized. Immunological characterization of *in vivo* VST expansion, including antigen-specific T-cell enumeration, was not available in this early hospital-exemption experience and will be prospectively incorporated into the formal ALLOVISTA trial protocol. All of this is planned to be taken into account during clinical trial development and in future ATMP-HE cases.

In conclusion, donor-derived VSTs, provided as a ATMP-HE, exhibited fast antiviral activity and an encouraging short-term safety profile in extremely vulnerable pediatric allo-HSCT patients with refractory viral infections. However, the advanced stage of the disease and competing post-transplant complications limited overall survival, emphasizing the need for earlier intervention, and to optimize patient selection, dosing, monitoring, and integration with other therapies.

## Data Availability

The original contributions presented in the study are included in the article/supplementary material. Further inquiries can be directed to the corresponding author.
